# Rational Engineering of AKR13B3 from *Devosia* A6-243 for Enhanced Aflatoxin B1 Degradation: A Dual Mechanism of Substrate Polarization and Tunnel Remodeling

**DOI:** 10.3390/ijms27167380

**Published:** 2026-08-18

**Authors:** Qingwei Jiang, Juan Shen, Zhanghu Chen, Xiaoqing Zhu, Caiyi Chen, Hao Zhu, Huibing Chi, Fengxia Lu, Ping Zhu

**Affiliations:** College of Food Science and Technology, Nanjing Agricultural University, Nanjing 210095, China

**Keywords:** AFB1-degrading enzymes, catalysis activity, rational design, structure analysis

## Abstract

Aflatoxin B1 (AFB1) is one of the most toxic mycotoxins, widely contaminating agricultural products and posing a serious threat to food safety and human health. Enzymatic degradation is considered a promising detoxification strategy due to its high efficiency, strong specificity, and lack of secondary pollution. AKR13B3, a member of the aldo-keto reductase family, possesses intrinsic catalytic activity for AFB1 degradation; however, its low natural activity severely limits practical application. In this study, the binding mode of the AKR13B3-NADPH complex with AFB1 was first determined using AlphaFold 3.0 and AutoDock Vina. Through interaction analysis, Trp102 and Asp41 were identified as key targets for enhancing catalytic activity. Following site-directed mutagenesis screening, two mutants, D41H and D41T, with significantly improved catalytic activity were obtained, exhibiting 52.32% and 46.44% higher activity than the wild-type enzyme, respectively. Three-dimensional structural simulation revealed that D41H and D41T form stable interactions with the carbonyl group on the lactone ring of AFB1, thereby polarizing the carbonyl group and reducing the activation energy of the reaction, ultimately enhancing catalytic activity. Substrate channel analysis demonstrated that, compared with the wild-type, the D41H and D41T mutants significantly increased the bottleneck radius of the substrate channel (by 25% and 22%, respectively) and shortened the channel length (by 23% and 33%, respectively), thereby partially relieving steric hindrance and diffusion limitations and improving catalytic efficiency. In summary, this study elucidates the molecular basis by which D41H and D41T enhance the catalytic activity of AKR13B3 toward AFB1 through the dual mechanisms of external/hydrogen bond catalysis and channel remodeling, providing an important theoretical foundation for the rational design and directed engineering of AFB1-degrading enzymes.

## 1. Introduction

Aflatoxin B1 (AFB1) is a highly toxic mycotoxin primarily produced by *Aspergillus flavus* and *Aspergillus parasiticus*, and is widely recognized as a Group 1 carcinogen [1,2]. It commonly pollutes staple foods like peanuts, corn and rice, which threatens global food safety and people’s health. Upon ingestion, AFB1 is metabolized by hepatic cytochrome P450 enzymes into the reactive intermediate AFB1-8,9-epoxide, which forms covalent adducts with DNA, activate cancer-related genes, which may finally result in liver cancer [3]. If organisms are exposed to AFB1 for a long time, they may have poor immunity, stunted growth and fetal abnormalities. It is essential to develop efficient methods to remove AFB1 in food and feed production [4,5].

Current strategies for AFB1 detoxification include physical adsorption, chemical degradation, and biological transformation [6,7]. Physical methods using activated carbon or clay minerals cannot selectively remove toxins. They only move toxins elsewhere instead of destroying them, which may cause secondary pollution [6,8]. Chemical approaches, including acid, alkali, or ozone treatment, often require harsh reaction conditions. Meanwhile, these treatments may produce harmful substances and damage the nutrition of food and feed [9]. In contrast, enzymatic biodegradation has emerged as the most promising approach due to its high specificity, mild reaction conditions, and environmental compatibility. In recent years, several enzymes capable of degrading AFB1 have been identified from diverse microbial sources, including laccases, peroxidases, and oxidoreductases [10]. Among these enzymes, aldo-keto reductases (AKRs) attract special attention. Certain AKRs rely on NADPH to reduce the carbonyl group in AFB1′s lactone ring and transform AFB1 into aflatoxicol (AFL), which has greatly decreased toxicity [11,12,13]. AKR13B3, a member of the AKR13B subfamily, was identified from *Devosia* sp. A6-243 and was named according to the unified nomenclature system of the AKR superfamily as the third functionally characterized member of this subfamily [14]. However, naturally occurring AKRs, including wild-type AKR13B3, generally show limited catalytic efficiency toward AFB1. Their low reaction rates compared with their natural substrates greatly limit their application in detoxification.

To overcome this limitation, enzyme engineering strategies have been extensively employed to improve catalytic performance. Traditional approaches include directed evolution and rational design [15]. Directed evolution imitates natural selection by introducing random mutations and screening the resulting variants to identify enzymes with improved properties [16]. Although this method has been widely used, it usually requires the construction and screening of large mutant libraries, which can be time-consuming and labor-intensive [17]. In comparison, rational design is a more efficient approach because it uses structural information to identify potential beneficial mutations before experiments [18]. By integrating structural analysis with computational tools, rational design enables the identification of key residues involved in substrate binding, catalysis, and conformational dynamics, thereby guiding site-directed mutagenesis with minimal experimental screening [19]. With the rapid advancement of computational biology, structure-guided rational design has become a powerful tool for enhancing enzyme activity. In particular, AlphaFold, a protein structure prediction tool developed by DeepMind, has greatly improved the ability to obtain reliable enzyme structures, providing an effective alternative for protein engineering research when experimentally resolved crystal structures are not available [20]. Several recent studies have successfully employed AlphaFold-based rational design to improve the catalytic efficiency, substrate specificity, and thermostability of various industrial enzymes, including cytochrome P450s, glycoside hydrolases, and nitrilases [21,22,23,24,25]. However, to date, the rational engineering of AKR-type enzymes for enhanced AFB1 degradation remains largely unexplored, representing a significant gap in the field. Critically, no experimental crystal structure of AKR13B3 has been resolved so far, which severely restricts the clarification of its substrate recognition mechanism and structural determinants for catalytic regulation. Although AKR13B3 has recently been engineered for the degradation of another mycotoxin, whether this enzyme can be rationally optimized for AFB1 degradation and what structural determinants govern substrate-specific catalytic enhancement remain unclear.

Previous studies have demonstrated that AKR13B3 can effectively degrade AFB1; however, its low catalytic activity severely limits its practical application in detoxification processes. In this study, we employed a rational design strategy combining AlphaFold-based structural simulation, molecular docking, and site-directed mutagenesis to improve the catalytic activity of AKR13B3. Key residues near the substrate-binding pocket were systematically mutated, and the resulting variants were characterized for their degradation efficiency against AFB1. Our work not only provides a highly active AFB1-detoxifying enzyme but also establishes a general computational framework for the rational engineering of mycotoxin-degrading enzymes.

## 2. Results and Discussion

### 2.1. Identification of Key Amino Acid Residues Affecting the Catalytic Activity of AKR13B3 for AFB1 Degradation

(1) AlphaFold-based prediction and analysis of the three-dimensional structure of AKR13B3 and its complex with coenzyme NADPH

**Figure 1:**
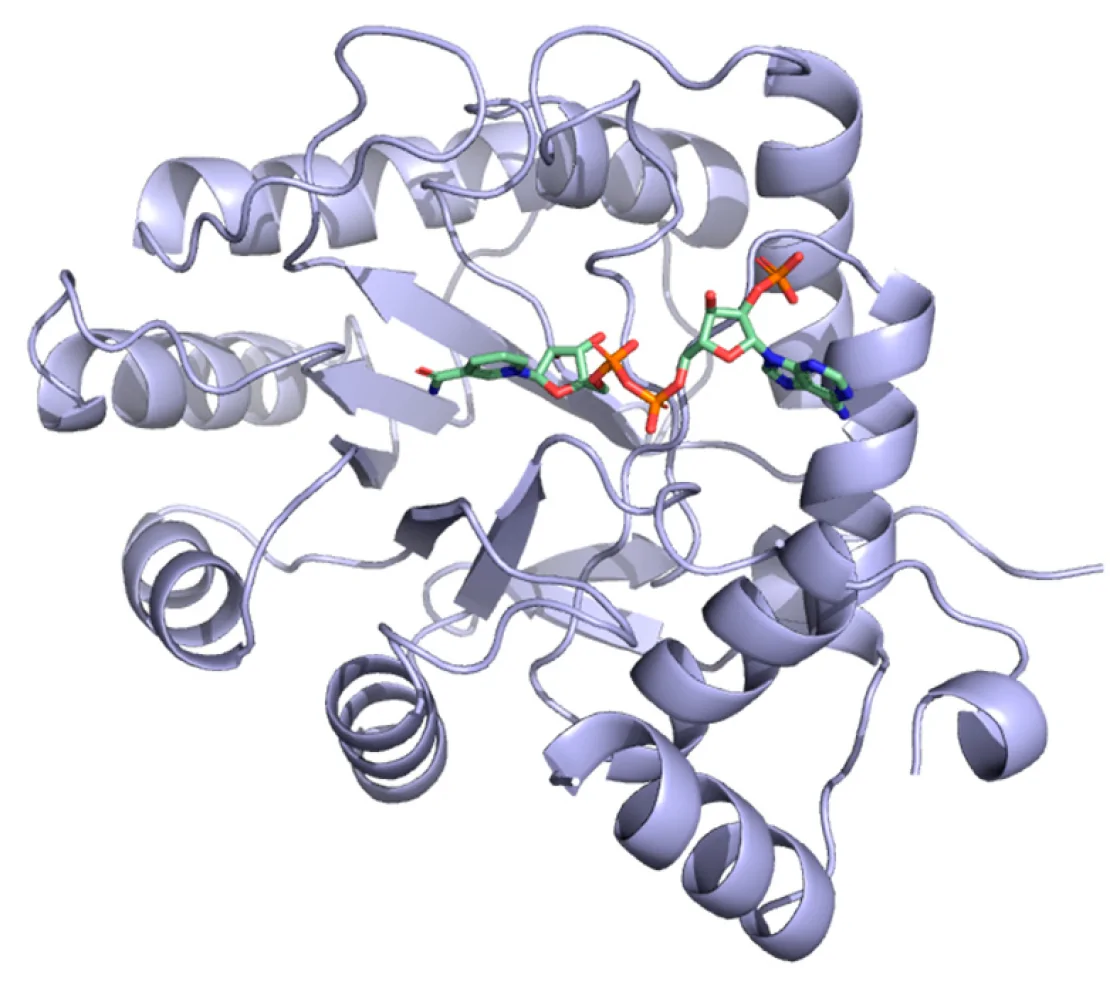
Three-dimensional structures of AKR13B3 protein and its complex with coenzyme NADPH.

The amino acid sequence of AKR13B3 was submitted to the AlphaFold 3.0 server as input. To predict its three-dimensional structure, the coenzyme NADPH was simultaneously included to perform co-folding simulations. After computation, five predictive models were generated. Based on confidence scores and structural rationality assessment, the top-ranked model (Model-0) was selected for subsequent molecular docking and functional analysis (Figure 1).

(2) Prediction and analysis of the three-dimensional structure of the AKR13B3-NADPH complex with the substrate AFB1 using AutoDock

To predict the binding mode between the AKR13B3-NADPH complex and the substrate AFB1, a semi-flexible molecular docking approach was employed. The receptor (AKR13B3-NADPH complex) was set as rigid, while the ligand AFB1 was treated as flexible. Docking calculations were performed using AutoDock Vina 1.1.2 software, generating a total of 100 docking conformations. The results were ranked according to binding affinity (kcal/mol), and the top nine conformations are listed in Table 1. Among these, Model 1 exhibited the lowest binding affinity of −7.6 kcal/mol, indicating the most stable binding. Models 2 and 3 showed binding affinities of −7.4 kcal/mol, followed by Models 4 to 9 with values of −7.3, −7.2, −7.1, −6.6, −6.6, and −6.3 kcal/mol, respectively. Negative binding affinity values indicate that the binding process is spontaneous, with lower values (i.e., greater absolute values) reflecting stronger protein-ligand interactions and better binding capability [26]. It is generally accepted that a binding affinity ≤−5.0 kcal/mol represents a relatively strong interaction [27].

**Figure 2:**
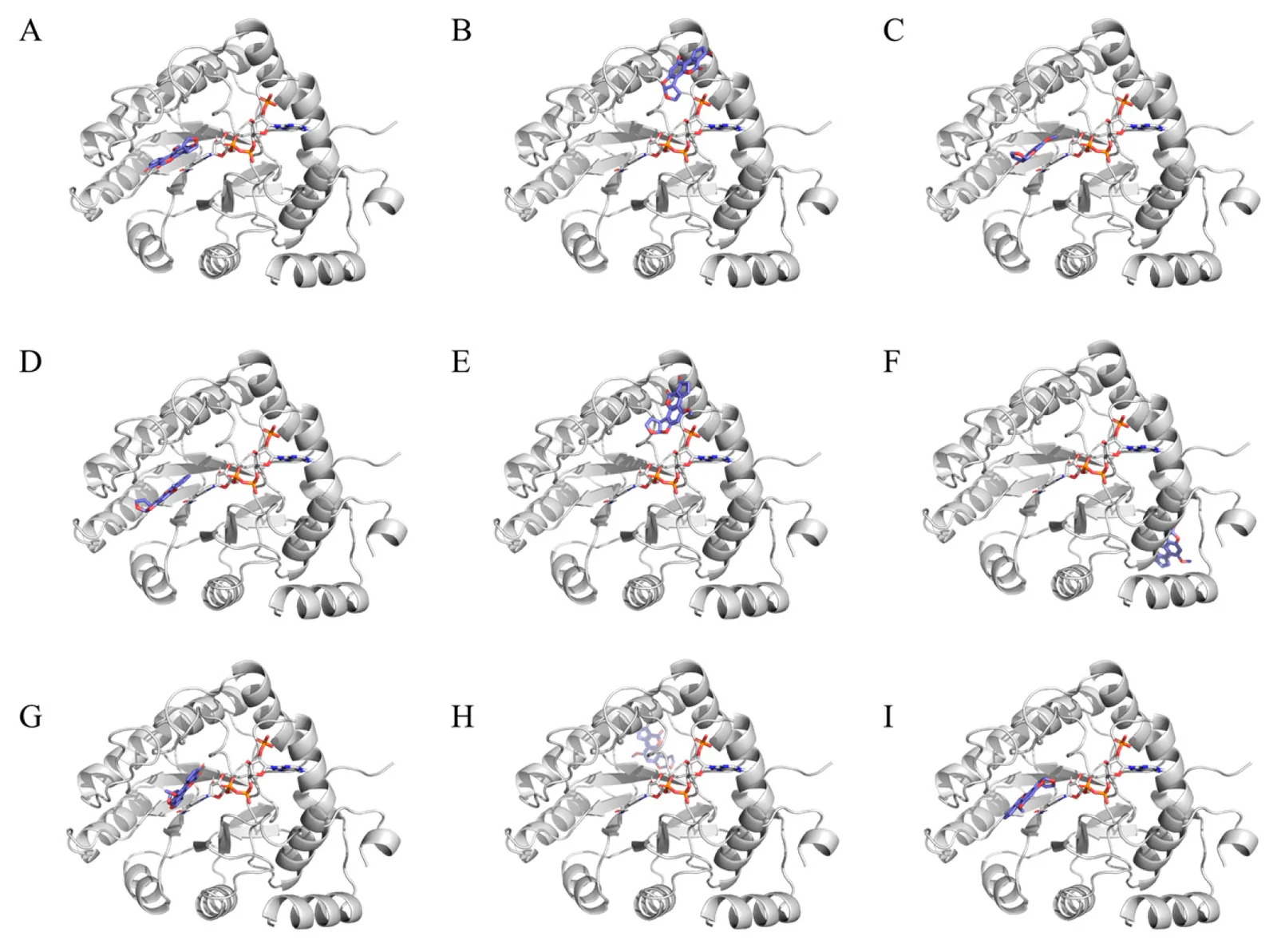
Three-dimensional structures of the AKR13B3-NADPH complex with the substrate AFB1. (**A**) 1. (**B**) Model 2; (**C**) Model 3; (**D**) Model 4; (**E**) Model 5; (**F**) Model 6; (**G**) Model 7; (**H**) Model 8; (**I**) Model 9.

Using the PyMOL visualization 3.1.1 software, the docking conformations of the AKR13B3-NADPH complex with the substrate AFB1 were analyzed in three dimensions. The binding position of the coenzyme NADPH was used as a reference to determine whether AFB1 was located within the protein’s active pocket. The results showed that in Models 1–5, 7, and 9, AFB1 stably bound in the vicinity of NADPH, residing within the predicted active site with a spatially reasonable orientation, consistent with typical enzyme-substrate binding characteristics (Figure 2). In contrast, in Models 6 and 8, AFB1 significantly deviated from the coenzyme position and was located far from the active center, suggesting these are likely false-positive conformations generated by docking. Therefore, subsequent interaction analyses (e.g., hydrogen bonds, hydrophobic interactions) were performed only for Models 1–5, 7, and 9.

(3) Identification of key amino acid residues affecting the catalytic activity of AKR13B3 for AFB1 degradation based on interaction analysis

**Figure 3:**
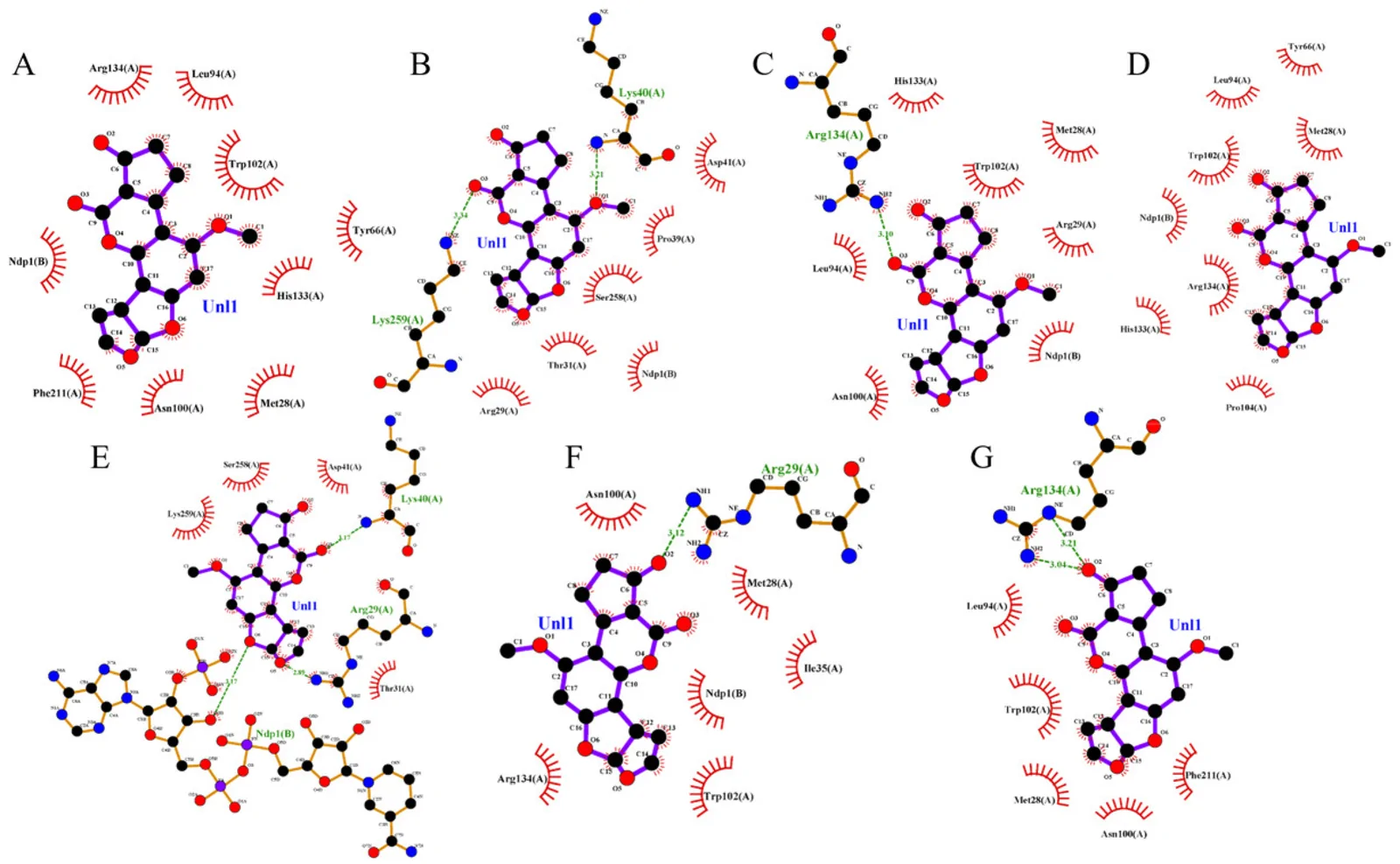
Interaction analysis of AFB1 with surrounding key amino acid residues in different docking models. (**A**) 1. (**B**) Model 2; (**C**) Model 3; (**D**) Model 4; (**E**) Model 5; (**F**) Model 7; (**G**) Model 9.

To enhance the catalytic activity of AKR13B3 for AFB1 degradation, this study screened key amino acid residues influencing its catalytic performance through interaction analysis. Previous studies have demonstrated that AKR family enzymes primarily catalyze the selective reduction of the carbonyl group (C=O) on the lactone ring of the AFB1 coumarin skeleton to a hydroxyl group (C–OH), producing AFL as the product [11,12]. Using LigPlus, the interactions between AFB1 and surrounding key amino acid residues in the docking models were analyzed (Figure 3). The results showed that in Model 1, the carbonyl group (C=O) on the lactone ring formed a hydrophobic interaction with Arg134. In Model 2, no interaction was observed. In Models 3 and 4, the carbonyl group formed a hydrophobic interaction with Trp102. In Model 5, it formed a hydrophobic interaction with Asp41. In Model 6, the carbonyl group formed a hydrogen bond with Arg29. In Model 7, it formed a hydrogen bond with Arg134. Given that hydrogen bonds contribute significantly to catalytic activity, and Arg29 and Arg134 are already engaged in hydrogen bonding with the substrate, their catalytic potential is relatively optimized. In contrast, Trp102 and Asp41 currently form only hydrophobic interactions; mutating them to amino acids capable of forming hydrogen bonds is expected to further improve the catalytic activity of AKR13B3. Therefore, excluding Arg29 and Arg134 which already form hydrogen bonds, Trp102 and Asp41 were ultimately identified as key targets for engineering to enhance catalytic activity.

### 2.2. Effects of Key Amino Acid Residues on the Catalytic Activity of AKR13B3 for AFB1 Degradation

**Figure 4:**
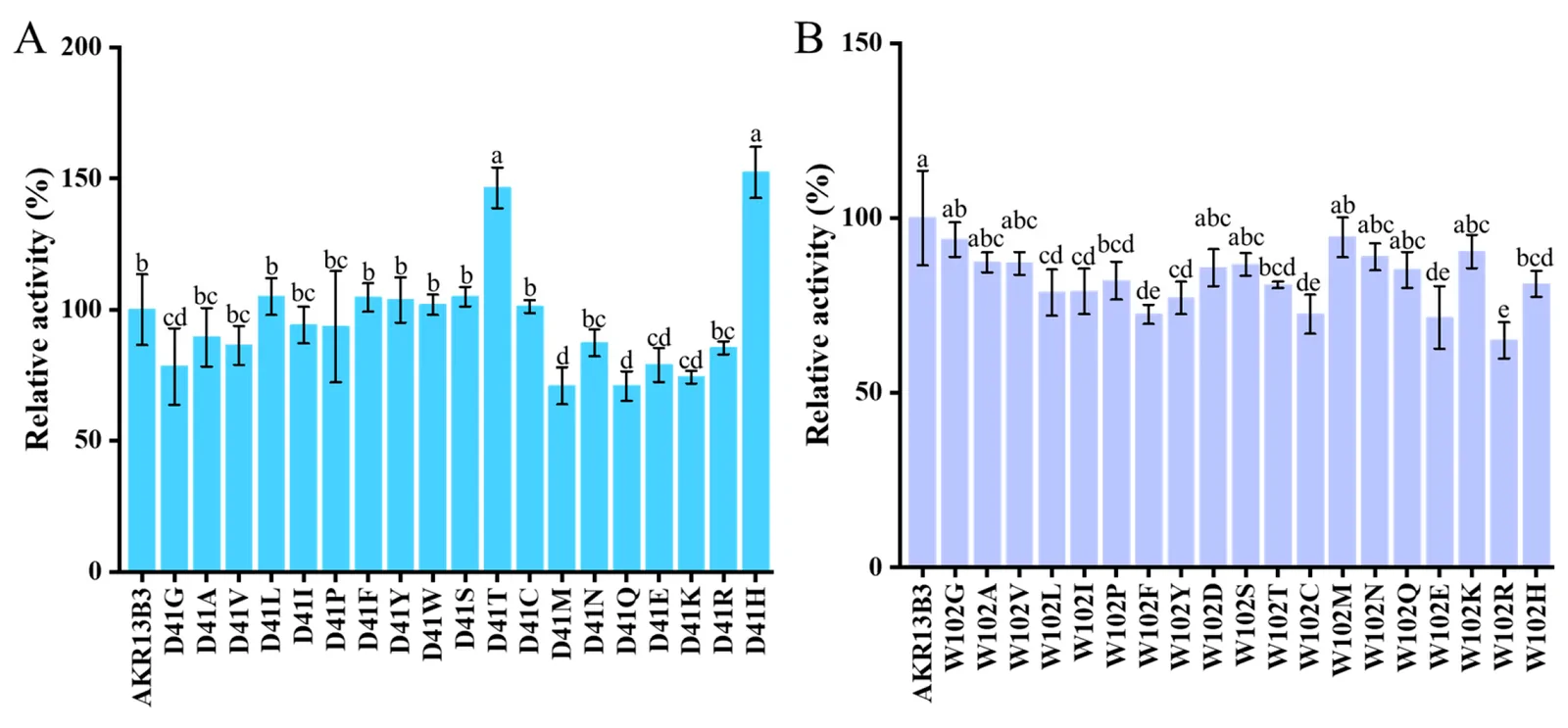
Relative activity of wild-type AKR13B3 and its site-saturation mutants at D41 (**A**) and W102 (**B**). Different lowercase letters indicate significant differences among groups, while the same letters denote no significant difference.

Purified wild‑type and mutant proteins at residue 41 (D41) and residue 102 (W102) were used for activity assays, and their relative activities were normalized to that of the wild‑type (WT) enzyme (set as 100%). The SDS‑PAGE profiles of these purified proteins are presented in Figure S1. The results showed that substitutions with different amino acids resulted in markedly distinct catalytic activities toward AFB1 degradation (Figure 4).

For the D41 site, among all 19 mutants, only D41H and D41T exhibited significantly enhanced catalytic activity, with relative activities reaching 152.32% and 146.44%, respectively, representing increases of approximately 52% and 46% compared with the wild-type. Mutants such as D41L, D41F, D41S, D41Y, D41W, and D41C maintained relative activities between 100% and 105%, showing comparable or slightly improved activity relative to the wild-type. Most of these mutants only maintained hydrophobic interactions with the substrate; however, some may have optimized the hydrophobic environment of the substrate-binding pocket through the introduction of hydrophobic side chains. The remaining mutants exhibited varying degrees of activity reduction. Among them, D41G (78.33%), D41E (78.95%), D41A (89.47%), D41V (86.38%), and D41R (85.45%) showed decreases of 5–22%, while D41M (70.90%), D41Q (70.90%), and D41K (74.30%) exhibited reductions of approximately 25–30%. These mutants failed to form effective stabilizing interactions with the substrate carbonyl group. Although some introduced charged or polar side chains, improper spatial orientation or an incompatible local microenvironment, and even disruption of the original active site conformation, likely contributed to the reduced catalytic efficiency.

For the W102 site, none of the 19 mutants exceeded the wild-type activity level. Among them, W102M (94.43%) and W102G (93.81%) showed the smallest decreases, maintaining approximately 94% of wild-type activity, suggesting that methionine and glycine can partially substitute for the function of tryptophan. Mutants such as W102K (90.40%), W102N (88.85%), W102A (87.31%), W102V (87.00%), and W102S (86.69%) exhibited activity reductions of approximately 10–15%. Mutants including W102D (85.76%), W102Q (85.14%), W102P (82.04%), W102H (81.11%), and W102T (80.80%) showed reductions of 15–20%. W102L (78.64%), W102I (78.95%), and W102Y (77.09%) exhibited reductions of approximately 22–23%. The most pronounced decreases were observed for W102F (72.45%), W102C (72.45%), W102E (71.52%), and W102R (65.02%), with W102R showing the lowest activity at only 65.02% of the wild-type level. Notably, previous molecular docking predicted that W102 forms hydrophobic interactions with the substrate, and it was hypothesized that mutating it to amino acids capable of hydrogen bonding might further enhance catalytic activity. However, the experimental data showed that none of the W102 mutants, including those introducing polar or charged residues (e.g., W102E, W102K, W102R, W102H, W102Q, W102N, W102S, W102T, W102D), exceeded wild-type activity. This result indicates that tryptophan at position 102 likely plays an irreplaceable structural role in the catalytic function of AKR13B3. Its large aromatic side chain is essential for maintaining the proper spatial configuration of the substrate-binding pocket and stabilizing the overall fold of the active site. Mutating it to other amino acids, even those capable of forming hydrogen bonds, disrupts the original active site architecture, leading to decreased catalytic efficiency.

Taken together, residue 41 is an effective target for enhancing catalytic activity, with D41H and D41T achieving activity breakthroughs by forming covalent or hydrogen bonds with the substrate carbonyl group. In contrast, residue 102 is not suitable as a target for activity enhancement, as its tryptophan residue plays an irreplaceable structural role. These findings further confirm that forming stable polar interactions with the substrate carbonyl group is the key molecular mechanism for improving the catalytic activity of AKR13B3.

### 2.3. Molecular Mechanism Underlying the Enhanced Catalytic Activity of AKR13B3 for AFB1 Degradation

(1) Static three-dimensional structural simulation analysis of the molecular mechanism

**Figure 5:**
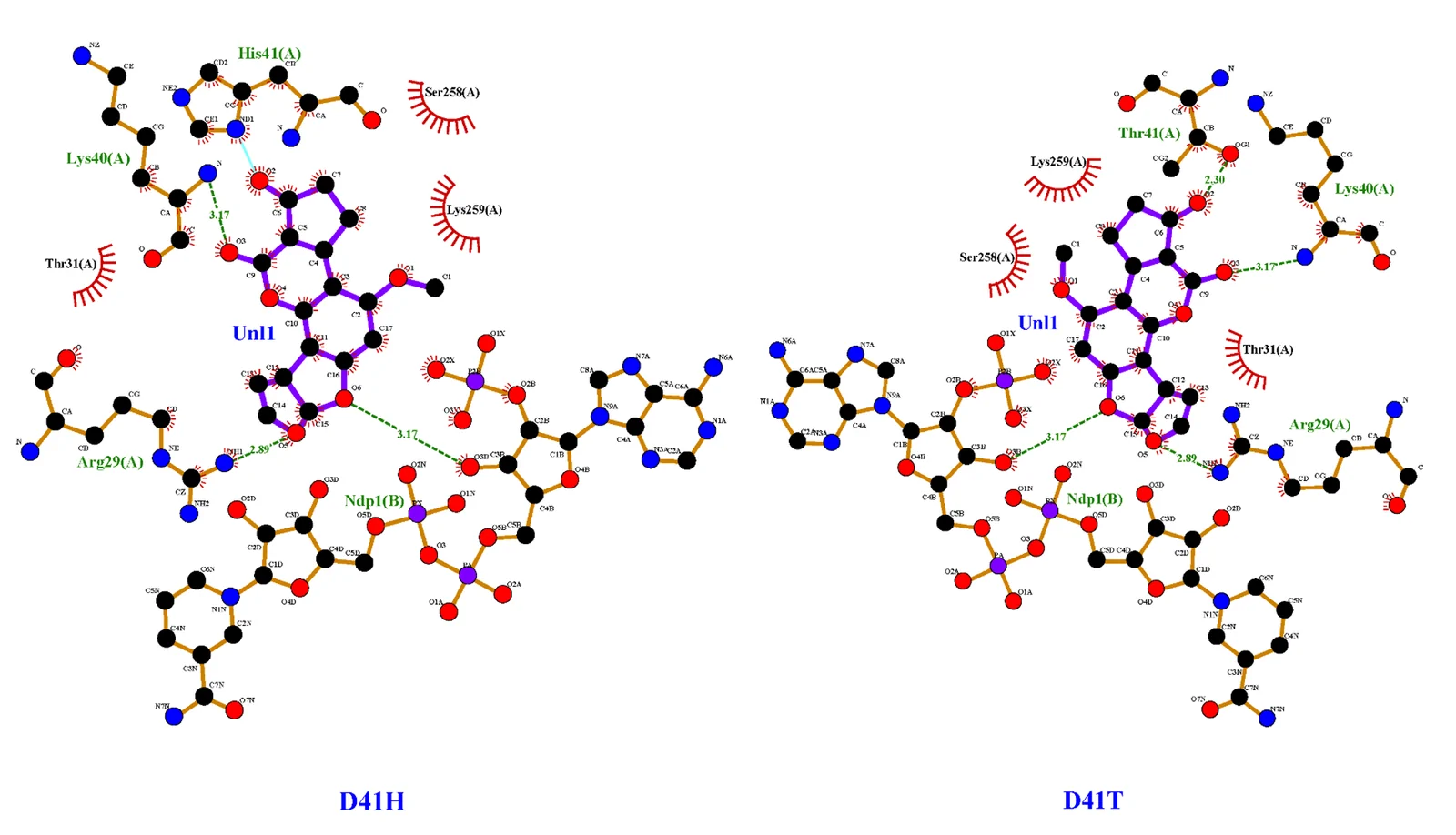
Interaction analysis of AFB1 with the substrate-binding pocket residues in D41H and D41T.

In wild-type AKR13B3, residue 41 is aspartic acid (D41). Interaction analysis revealed that this residue failed to form any effective stabilizing interaction with the carbonyl group (C=O) on the lactone ring of the substrate AFB1. This indicates that during catalysis, the carbonyl group remains in a relatively unactivated state, requiring the reaction to overcome a high activation energy barrier. Specifically, although aspartic acid possesses a negatively charged side chain, the spatial orientation of its carboxyl group or the local microenvironment may not favor the formation of an ideal hydrogen bond or electrostatic interaction with the substrate carbonyl group, resulting in a very limited catalytic contribution from this site. Furthermore, systematic analysis of 19 mutants showed that substituting D41 with 17 other amino acids (A, C, E, F, G, I, K, L, M, N, P, Q, R, S, V, W, Y) only maintained hydrophobic interactions and failed to introduce chemical groups capable of directly stabilizing the substrate C=O (Figure 5 and Figure S2). This explains why these mutations did not enhance catalytic activity—they merely altered the hydrophobic environment of the binding channel without affecting the chemical step itself.

When residue 41 was mutated from aspartic acid to histidine (D41H), the introduced histidine side chain—the imidazole ring—possesses unique nucleophilic properties. Interaction analysis showed that histidine forms an external bond with the carbonyl group (C=O) on the lactone ring of AFB1. The formation of this interaction has profound catalytic implications. The imidazole nitrogen of histidine likely acts as a nucleophile, attacking the carbonyl carbon of the substrate lactone ring to form an intermediate. In this covalent adduct, the C=O bond of the substrate lactone ring becomes significantly polarized: the carbonyl carbon acquires increased positive charge, while the carbonyl oxygen gains enhanced negative charge. This polarization effect greatly reduces the activation energy required for subsequent nucleophilic attack (e.g., by water molecules or nucleophiles provided by the coenzyme) on the carbonyl carbon. From a catalytic mechanism perspective, D41H introduces a strategy akin to “covalent catalysis.” This mechanism is commonly observed in hydrolase families such as serine and cysteine proteases, with the core advantage of decomposing a high-energy-barrier step in the reaction pathway into several lower-barrier steps, thereby significantly increasing the reaction rate [28,29]. In the AFB1 degradation reaction catalyzed by AKR13B3, D41H directly accelerates the cleavage of the C=O bond on the substrate lactone ring through this mechanism.

When residue 41 was mutated from aspartic acid to threonine (D41T), the introduced threonine side chain contains a hydroxyl group (-OH). Interaction analysis revealed that this hydroxyl group forms a hydrogen bond with the carbonyl group (C=O) on the lactone ring of AFB1. The hydroxyl group of threonine acts as a hydrogen bond donor. After forming a hydrogen bond with the oxygen atom of the substrate C=O, the carbonyl group is polarized via electrostatic effects: electron density on the oxygen atom is partially transferred to the hydroxyl group, resulting in increased positive charge on the carbonyl carbon. This polarization makes the carbonyl carbon more susceptible to nucleophilic attack, thereby lowering the reaction activation energy. The effect of hydrogen bond catalysis is relatively weaker, but its advantages include strong reversibility, minimal perturbation of the active site conformation, and a low risk of irreversible side reactions. In the D41T mutant, threonine provides a mild but effective transition-state stabilization mechanism, moderately improving catalytic efficiency without introducing the risk of covalent modification.

It is worth emphasizing that among the 19 mutants, only histidine (H) and threonine (T) were able to form direct stabilizing interactions with the substrate C=O—histidine forming an external bond and threonine forming a hydrogen bond. The other 17 mutants only maintained hydrophobic interactions, failing to introduce any chemical groups with nucleophilic or hydrogen-bond-donating capabilities toward the carbonyl group. This selectivity reveals the critical role of residue 41 in catalysis: it requires not only appropriate spatial positioning but also specific chemical properties. The imidazole ring of histidine provides nucleophilicity, while the hydroxyl group of threonine offers hydrogen bond donor capability. Other polar amino acids (such as asparagine, glutamine, and serine), despite possessing polar side chains, likely fail to form effective interactions with the substrate C=O due to constraints in spatial orientation, pKa, or the local microenvironment.

(2) Substrate channel analysis reveals the structural basis for enhanced catalytic activity of AKR13B3 mutants

To investigate the molecular mechanism underlying the improved catalytic activity of the D41H and D41T mutants, this study systematically analyzed the substrate channels of wild-type AKR13B3 and its two mutants using CAVER WEB v2.0 (https://loschmidt.chemi.muni.cz/caverweb/). Approximately 500 snapshots were examined, and key channel parameters including bottleneck radius, length, curvature, and throughput were compared. The results showed that both mutants exhibited significant alterations in channel characteristics compared with the wild type (Table 2).

In terms of bottleneck radius, wild-type AKR13B3 had an average bottleneck radius of 2.36 Å. The D41H mutant significantly increased this radius to 2.96 Å, representing an increase of approximately 25%, while the D41T mutant increased it to 2.87 Å, an increase of approximately 22%. These results indicate that both mutations effectively widened the narrowest constriction of the channel. Regarding channel length, the wild type exhibited an average length of 4.51 Å. The D41H mutant shortened the length to 3.47 Å, a reduction of approximately 23%, while the D41T mutant further reduced it to 3.01 Å, a reduction of up to 33%. This suggests that the mutations not only widened the channel but also significantly shortened the diffusion distance from the protein surface to the active site. In terms of curvature, the wild type showed a curvature value of 1.24, compared with 1.23 for D41H and 1.20 for D41T, indicating no substantial difference among the three. This suggests that the mutations did not alter the overall bending pattern of the channel, and the geometric trajectory remained largely unchanged. Regarding throughput, the wild type exhibited a value of 0.88, whereas D41H and D41T increased to 0.92 and 0.94, respectively, corresponding to improvements of 4.5% and 6.8%. Although the bottleneck radius and channel length both showed significant improvements, the increase in throughput was relatively modest. Furthermore, comparing the two mutants, D41H exhibited a slightly larger increase in bottleneck radius than D41T, while D41T showed a more pronounced shortening of channel length and a slightly higher throughput. These results indicate that the D41H and D41T mutations partially relieve the diffusion limitations present in the wild-type enzyme by reshaping the geometry of the substrate channel, thereby enhancing catalytic activity.

The mutation-induced increase in bottleneck radius relieves steric hindrance, thereby improving catalytic activity [30]. The bottleneck radius of wild-type AKR13B3 is only 2.36 Å, a size that can barely accommodate water molecules or very small ions. For most organic substrates, the channel remains narrow or even closed in the majority of dynamic conformations. Substrate entry and product release are severely restricted by steric hindrance, making diffusion resistance a rate-limiting factor in catalysis [31]. The D41H and D41T mutations increased the bottleneck radius to 2.96 Å and 2.87 Å, respectively. An increase of 0.5–0.6 Å, though seemingly small at the molecular scale, represents a substantial qualitative improvement. This change effectively “opens up” the narrowest constriction, allowing substrate intermediates or product molecules that previously could not pass smoothly to now have the physical possibility of traversing the channel. The relief of steric hindrance directly reduces diffusion resistance during substrate entry into the active site and product release [32]. From a structural mechanism perspective, residue 41 is likely located at the bottleneck region of the channel. Aspartic acid has a small, negatively charged side chain. When mutated to histidine or threonine, the imidazole ring of histidine, being relatively bulky, may physically push the channel wall outward. Although threonine has a smaller side chain, its hydroxyl group may indirectly influence channel wall conformation by remodeling the local hydrogen bond network. Both mutations achieved significant increases in bottleneck radius, with D41H exhibiting a slightly greater widening effect due to its larger side-chain volume.

The mutation-induced shortening of channel length optimizes the diffusion path, thereby improving catalytic activity. The average channel length of the wild type is 4.51 Å, whereas D41H and D41T shorten it to 3.47 Å and 3.01 Å, respectively, representing reductions of 23–33%. Channel length directly determines the migration distance that substrates must travel from the protein surface to the active site, as well as the distance for product release from the active site back to the surface [33]. Shorter channel length reduces diffusion time and accelerates mass exchange [34]. The significant shortening of the channel length in the mutants means that the “journey” of substrates and products through the channel is substantially curtailed, further accelerating mass transport during the catalytic cycle [35,36]. Notably, curvature remained essentially unchanged before and after mutation, indicating that the overall trajectory of the channel was preserved, and the length reduction resulted from localized optimization along the same channel.

It is noteworthy that despite a 22–25% increase in bottleneck radius and a 23–33% decrease in channel length, throughput increased by only 4.5–6.8%. This apparent “dose-effect discrepancy” warrants further discussion. The most likely explanation is that the throughput algorithm in CAVER assigns substantial weight to the “open frequency” of the channel across simulation snapshots. Although the mutations made the channel wider and shorter when in an open state, they did not significantly increase the proportion of time during which the channel remains open. In other words, the mutations improved the “quality of the open state” but did not alter the “probability of opening.” Alternatively, other unmeasured rate-limiting factors may exist, such as electrostatic interactions between the substrate and channel wall, hydrophobic matching, or transient blocking effects of local residues during molecular dynamics simulations [37,38]. Another possibility is that the chemical step of product dissociation from the active site remains partially rate-limiting; even if the channel is fully unobstructed, the overall catalytic rate may not achieve a proportional increase.

Furthermore, D41H exhibited a slightly greater increase in bottleneck radius than D41T, whereas D41T showed a more pronounced reduction in channel length and a marginally higher throughput. This difference suggests that the two mutations may enhance catalytic activity through somewhat distinct structural mechanisms: D41H focuses on “widening more,” using the bulky histidine side chain to directly expand the bottleneck, whereas D41T emphasizes “shortening the path,” allowing the smaller threonine side chain to make the channel trajectory more compact. From a throughput perspective, the contribution of path shortening may slightly outweigh that of bottleneck widening, providing valuable insights for future rational design. In summary, the D41H and D41T mutations partially relieve the diffusion limitations in wild-type AKR13B3 by synergistically increasing bottleneck radius and shortening channel length, thereby enhancing catalytic activity. However, the modest throughput increase suggests that open frequency and other rate-limiting factors remain potential targets for further optimization of this enzyme’s catalytic efficiency. Future efforts may focus on site-directed mutagenesis to fine-tune the side-chain properties of bottleneck residues or combine directed evolution strategies to increase channel opening probability, aiming for greater improvements in catalytic activity.

**Table 2:** Channel characteristics of wild-type and mutant AKR13B3.

Enzyme	Snapshots	Bottleneck Radius (Å)	Max BR (Å)	Length (Å)	Curvature	Throughput
AKR13B3	501	2.36 ± 0.57	4.45	4.51 ± 2.24	1.24 ± 0.26	0.88 ± 0.06
D41H	496	2.96 ± 0.75	5.11	3.47 ± 2.13	1.23 ± 0.27	0.92 ± 0.05
D41T	495	2.87 ± 0.66	4.4	3.01 ± 1.7	1.2 ± 0.26	0.94 ± 0.04

Although AlphaFold3 has become a useful tool for protein structure prediction and enzyme engineering, the predicted models cannot replace experimentally determined structures. In this study, the AlphaFold3 model of AKR13B3 was used as a reference to identify residues potentially involved in AFB1 recognition and to assist in selecting mutation sites. By combining structural analysis, molecular interaction analysis, and experimental characterization, we identified D41 as an important residue influencing the catalytic activity of AKR13B3. Nevertheless, obtaining an experimentally resolved structure of AKR13B3, especially a complex structure with AFB1 and cofactors, would provide stronger evidence for understanding substrate binding, catalytic processes, and the structural effects caused by mutations. Such structural information would help further evaluate the proposed roles of substrate carbonyl polarization and substrate channel changes in activity enhancement and would be valuable for future engineering of AKR13B3.

Although this study focused on improving the AFB1-degrading activity of AKR13B3, the structure-guided engineering workflow established here may provide useful insights for future optimization of other mycotoxin-degrading enzymes. This strategy could potentially facilitate the identification of key residues involved in substrate recognition and catalytic enhancement. However, the applicability of this approach to other mycotoxin-degrading systems requires further investigation and validation. Future studies evaluating the substrate spectrum of engineered AKR13B3 variants toward other aflatoxin derivatives (e.g., AFB2, AFG1, AFG2, and AFM1) will also help clarify their catalytic versatility and application potential.

## 3. Methods and Materials

### 3.1. Strains, Chemicals, and Reagents

The gene encoding AKR13B3 was synthesized and cloned into the pET-28a(+) expression vector, and the recombinant plasmid was transformed into Escherichia coli BL21(DE3) competent cells for protein expression. *E. coli* BL21(DE3) Competent Cells were purchased from Sangon Biotech Co., Ltd. (Shanghai, China). The 2 × Phanta Max Master Mix for PCR was obtained from Vazyme Biotech Co., Ltd. (Nanjing, China), and QuickCut™ DpnI for plasmid template digestion was purchased from Takara Biomedical Technology Co., Ltd. (Beijing, China). Gel Extraction Kit, One Step Seamless Cloning Kit, and PurePlasmid Mini Kit were all sourced from Jiangsu CoWin Biotech Co., Ltd. (Taizhou, China). All constructed plasmids were sequenced at Jiangsu CoWin Biotech Co., Ltd. For protein purification, High Affinity Ni-Charged Resin FF was purchased from Nanjing GeneScript Biotech Co., Ltd. (Nanjing, China). Unstained Protein Molecular Weight Marker 26610 were purchased from Thermo Fisher Scientific (Waltham, MA, USA). AFB1 were purchased from Pribolab (Qingdao, China). HPLC-grade Acetonitrile used for AFB1 detection were purchased from Anhui Tedia High Purity Solvents Co., Ltd. (Anqing, China). All other chemicals used in this study were of analytical grade and commercially available.

### 3.2. Three-Dimensional Structural Simulation of AKR13B3 and Its Mutants

The amino acid sequences of wild-type AKR13B3 and its mutants were submitted to the AlphaFold 3.0 online server (https://alphafoldserver.com) for three-dimensional structure prediction [20]. For co-factor binding simulation, NADPH was simultaneously selected to perform co-folding simulations. Among the five predictive models generated, the top-ranked model (Model-0) with the highest confidence score was selected for subsequent molecular docking and structural analysis.

### 3.3. Molecular Docking of the AKR13B3-NADPH Complex with AFB1

To predict the binding mode between the AKR13B3-NADPH complex and the substrate AFB1, a semi-flexible molecular docking approach was employed using AutoDock Vina software [39]. The receptor (AKR13B3-NADPH complex) was set as rigid, while the ligand AFB1 was treated as flexible. A total of 100 docking conformations were generated. The results were ranked according to binding affinity (kcal/mol). The docking poses were visualized and analyzed using PyMOL software (https://pymol.org).

### 3.4. Identification of Key Residues for Enhancing Catalytic Activity

Based on the docking results, interactions between AFB1 and surrounding amino acid residues were analyzed using LigPlus v2.3.1 software [40]. Residues forming hydrogen bonds or hydrophobic interactions with the carbonyl group (C=O) on the lactone ring of AFB1 were identified as candidates. Among these, residues that already formed hydrogen bonds were considered relatively optimized, while those that formed only hydrophobic interactions were selected as targets for site-directed mutagenesis to introduce hydrogen bond donor or nucleophilic groups.

### 3.5. Site-Directed Mutagenesis

Site-directed mutagenesis was performed to introduce substitutions at selected residues (D41 and W102). Primers used in this study are listed in Supplementary Table S1. The PCR reaction system had a total volume of 25 μL, consisting of 2 ng of template DNA, 0.4 μM of both forward and reverse primers, 12.5 μL of 2 × Phanta Max Master Mix, with the remaining volume made up with ddH_2_O. The PCR cycling conditions were as follows: initial denaturation at 95 °C for 5 min; denaturation at 95 °C for 30 s, annealing at 58 °C for 30 s, and extension at 72 °C for 4 min, for a total of 30 cycles; followed by a final extension at 72 °C for 5 min. The amplification product containing the original template plasmid was digested with QuickCut™ DpnI at 37 °C for 1 h to avoid false-positive transformants. After purification of the target fragment using the Gel Extraction Kit, the linear DNA was circularized using the One Step Seamless Cloning Kit at 50 °C for 20 min. Finally, transformation and sequencing were performed to confirm successful construction of each mutant.

### 3.6. Heterologous Expression and Purification of Wild-Type and Mutant Enzymes

The constructed plasmids were retransformed into *E. coli* BL21(DE3) competent cells. *E. coli* strains carrying wild-type or mutant gene expression vectors were inoculated into 5 mL of LB medium containing 50 μg/mL kanamycin and cultured overnight at 37 °C with shaking at 200 rpm. The next day, 1 mL of the bacterial culture was transferred into 100 mL of LB medium and incubated at 37 °C with shaking at 200 rpm. When the OD_600_ reached 0.6–0.8, 0.2 mM IPTG was added, and the culture was incubated at 16 °C with shaking at 200 rpm for 16 h.

The fermented bacterial culture was then centrifuged at 8000 rpm for 3 min, and the supernatant was discarded. Five mL of disruption buffer (20 mM Tris-HCl, 200 mM NaCl, pH 8.0) was added, and the cells were sonicated for 5 min at 200 W. The resulting cell lysate was centrifuged at 8000 rpm for 20 min, and the supernatant was collected. This was filtered through a 0.22 μm filter and subsequently purified using High Affinity Ni-Charged Resin FF. Finally, the protein’s molecular weight and purity were confirmed by SDS-PAGE.

### 3.7. Assays for the Degradation Rate of AFB1

The catalytic activity of wild-type AKR13B3 and its mutants was measured by the degradation rate of AFB1. The reaction system consisted of 250 μg/mL of purified protein, 0.2 mM NADPH, 1 μM AFB1, and 50 mM Tris-HCl buffer (pH 7.5), and was incubated at 37 °C for 24 h. After incubation, the reaction was terminated by adding an equal volume of methanol. The mixture was filtered through a 0.22 μm nylon membrane, and the filtrate was analyzed by high-performance liquid chromatography (HPLC). The chromatographic column was a ZORBAX Eclipse Plus C18 (4.6 × 150 mm, 5 μm). Elution was carried out with a mobile phase consisting of water/Acetonitrile (70:30, *v*/*v*) at a flow rate of 0.2 mL/min for 20 min, and the detection wavelength was 365 nm for AFB1. The degradation efficiency was calculated as the percentage of AFB1 removed after enzymatic treatment: degradation efficiency (%) = [(C_1_ − C_2_)/C_1_] × 100%, where C_1_ represents the initial AFB1 concentration and C_2_ represents the residual AFB1 concentration. The relative activity of the wild-type enzyme was normalized to 100% for comparative purposes.

### 3.8. Substrate Channel Analysis

Substrate channel analysis was performed using CAVER 3.0 software based on the predicted three-dimensional structures of wild-type AKR13B3 and its mutants [41]. Key channel parameters, including bottleneck radius, channel length, curvature, and throughput, were calculated and compared among wild-type, D41H, and D41T.

### 3.9. Statistical Analysis

All experiments were performed with at least three independent replicates. Data are presented as the mean ± standard deviation (SD). Statistical comparisons were performed using Student’s *t*-test or one-way ANOVA followed by Tukey’s post hoc test, with a significance threshold of *p* < 0.05. Figures were prepared and laid out using Origin 2022.

## 4. Conclusions

In summary, based on a structure-guided rational design strategy combining molecular docking, interaction analysis, and site-directed mutagenesis, this study successfully obtained two AKR13B3 mutants, D41H and D41T, with significantly enhanced catalytic activity toward AFB1. To the best of our knowledge, this is the first report on the rational engineering of an AKR13B family member through single-point mutation to improve AFB1 degradation activity. Further three-dimensional structural simulation and substrate channel analysis revealed the underlying mechanism by which D41H and D41T synergistically enhance catalytic activity. Following the introduction of histidine or threonine at position 41, the mutants formed stable interactions with the carbonyl group on the lactone ring of AFB1, thereby polarizing the carbonyl group and reducing the activation energy of the reaction. Building upon this catalytically favorable framework, the mutants also achieved geometric remodeling of the substrate channel—characterized by a significantly enlarged bottleneck radius and a markedly shortened channel length—which partially relieved steric hindrance and diffusion limitations, synergistically contributing to the improved catalytic activity. This study provides an effective engineering strategy and mechanistic insights for enhancing the catalytic activity of AKR13B3 toward AFB1 degradation, holding great promise for applications in food safety and mycotoxin detoxification.

## Figures and Tables

**Table 1:** Molecular docking scores of the AKR13B3-NADPH complex with the substrate AFB1.

Model	Affinity (kcal/mol)
1	−7.6
2	−7.4
3	−7.4
4	−7.3
5	−7.2
6	−7.1
7	−6.6
8	−6.6
9	−6.3

## Data Availability

The original contributions presented in this study are included in the article/Supplementary Material. Further inquiries can be directed to the corresponding authors.

## Supplementary Materials

The following supporting information can be downloaded at: https://www.mdpi.com/article/10.3390/ijms27167380/s1.
