# Supplementary material for: Rational Engineering of AKR13B3 from Devosia A6-243 for Enhanced Aflatoxin B1 Degradation: A Dual Mechanism of Substrate Polarization and Tunnel Remodeling
Source: Int J Mol Sci. 2026 Aug 18;27(16):7380. doi: 10.3390/ijms27167380 (PMC13513513; doi:10.3390/ijms27167380)
Supplement: Supplementary file 1 [file ijms-27-07380-s001.zip › ijms-4422503-supplementary.pdf]

Supplementary Material For:

Rational Engineering of AKR13B3 from *Devosia* A6-243 for Enhanced Aflatoxin B1 Degradation: A Dual Mechanism of Substrate Polarization and Tunnel Remodeling

Table S1. The primers used in this study.

Primer	Sequence
D41G-F	ctggggtccgcccaagGGAcgtgacgaagccatt
D41A-F	ctggggtccgcccaagGCAcgtgacgaagccatt
D41V-F	ctggggtccgcccaagGTTcgtgacgaagccatt
D41L-F	ctggggtccgcccaagCTTcgtgacgaagccatt
D41I-F	ctggggtccgcccaagATTcgtgacgaagccatt
D41P-F	ctggggtccgcccaagCCAcgtgacgaagccatt
D41F-F	ctggggtccgcccaagTTTcgtgacgaagccatt
D41Y-F	ctggggtccgcccaagTATcgtgacgaagccatt
D41W-F	ctggggtccgcccaagTGGcgtgacgaagccatt
D41S-F	ctggggtccgcccaagTCTcgtgacgaagccatt
D41T-F	ctggggtccgcccaagACTcgtgacgaagccatt
D41C-F	ctggggtccgcccaagTGTcgtgacgaagccatt
D41M-F	ctggggtccgcccaagATGcgtgacgaagccatt
D41N-F	ctggggtccgcccaagAATcgtgacgaagccatt
D41Q-F	ctggggtccgcccaagCAAcgtgacgaagccatt
D41E-F	ctggggtccgcccaagGAAcgtgacgaagccatt
D41K-F	ctggggtccgcccaagAAAcgtgacgaagccatt
D41R-F	ctggggtccgcccaagCGCcgtgacgaagccatt
D41H-F	ctggggtccgcccaagCACcgtgacgaagccatt
D41-R	cttgggcggacccca

Primer	Sequence
W102G-F	cagcggtcccaatcaaGGTccgccgctggggcgt
W102A-F	cagcggtcccaatcaaGCTccgccgctggggcgt
W102V-F	cagcggtcccaatcaaGTTccgccgctggggcgt
W102L-F	cagcggtcccaatcaaTTAccgccgctggggcgt
W102I-F	cagcggtcccaatcaaATTccgccgctggggcgt
W102P-F	cagcggtcccaatcaaCCTccgccgctggggcgt
W102F-F	cagcggtcccaatcaaTTTccgccgctggggcgt
W102Y-F	cagcggtcccaatcaaTATccgccgctggggcgt
W102S-F	cagcggtcccaatcaaTCTccgccgctggggcgt
W102T-F	cagcggtcccaatcaaACTccgccgctggggcgt
W102C-F	cagcggtcccaatcaaTGTccgccgctggggcgt
W102M-F	cagcggtcccaatcaaATGccgccgctggggcgt
W102N-F	cagcggtcccaatcaaAATccgccgctggggcgt
W102Q-F	cagcggtcccaatcaaCAAccgccgctggggcgt
W102D-F	cagcggtcccaatcaaGATccgccgctggggcgt
W102E-F	cagcggtcccaatcaaGAAccgccgctggggcgt
W102K-F	cagcggtcccaatcaaAAAccgccgctggggcgt
W102R-F	cagcggtcccaatcaaAGAccgccgctggggcgt
W102H-F	cagcggtcccaatcaaCATccgccgctggggcgt
W102-R	ttgattgggaccgct

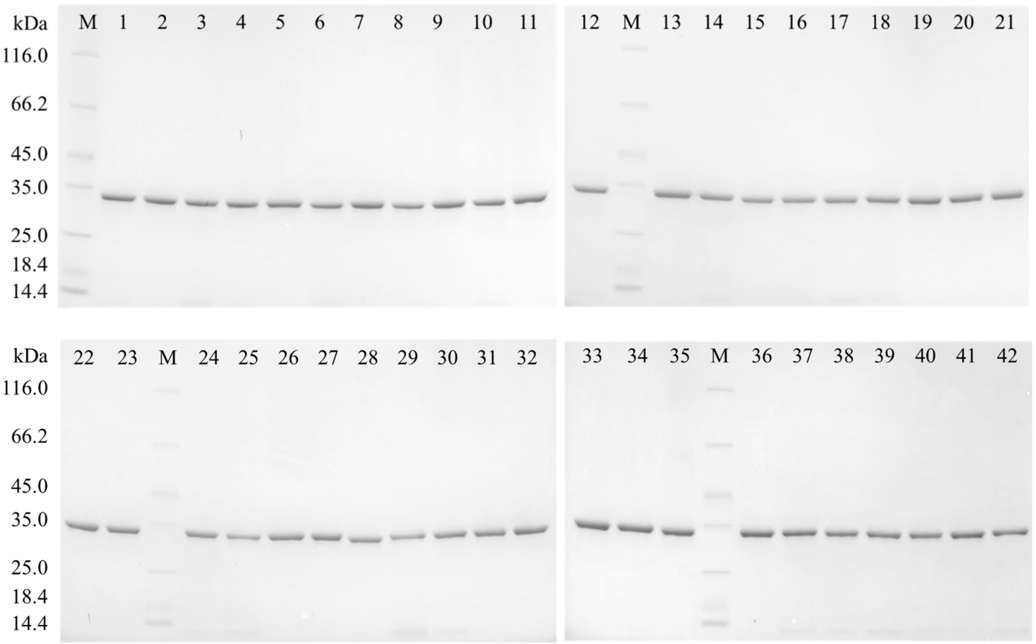

Fig. S1. SDS-PAGE analysis of wild type and its mutant enzymes.

M: Protein Marker; Lane 1: AKR13B3; Lane 2:D41G; Lane 3: D41A; Lane 4: D41V; Lane 5: D41L; Lane 6: D41I; Lane 7:D41P; Lane 8: D41F; Lane 9: D41Y; Lane 10: D41W; Lane 11: D41S; Lane 12: D41T; Lane 13: D41C; Lane 14: D41M; Lane 15: D41N; Lane 16:D41Q; Lane 17: D41E; Lane 18:D41K; Lane 19: D41R; Lane 20: D41H; Lane 21: AKR13B3; Lane 22: AKR13B3; Lane 23:W102G; Lane 24: W102A; Lane 25: W102V; Lane 26: W102L; Lane 27: W102I; Lane 28:W102P; Lane 29: W102F; Lane 30: W102Y; Lane 31: W102D; Lane 32: W102S; Lane 33: W102T; Lane 34: W102C; Lane 35: W102M; Lane 36: W102N; Lane 37:W102Q; Lane 38: W102E; Lane 39:W102K; Lane 40: W102R; Lane 41: W102H; Lane 42: AKR13B3.

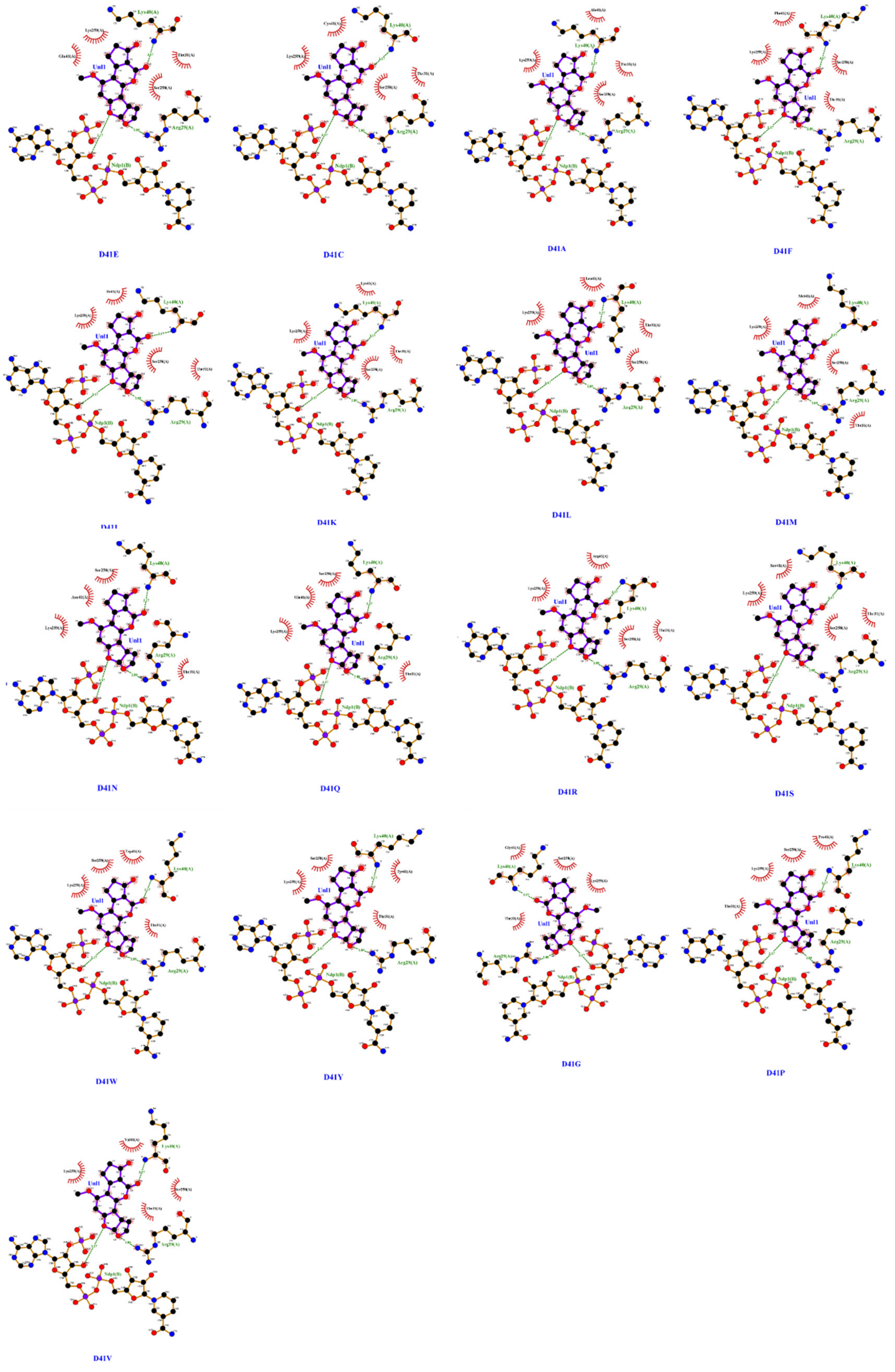

Fig. S2. Interaction analysis of AFB1 with the substrate-binding pocket residues in D41 mutants.
